# A Combined Approach Using Strip Grafts and Xenogenic Dermal Matrix for Peri-Implant Keratinized Mucosa Augmentation in the Mandible: A Case Series

**DOI:** 10.3390/biomedicines13040806

**Published:** 2025-03-27

**Authors:** Xinda Li, Dániel Palkovics, Péter Windisch, Željka Perić Kačarević, Attila Horváth

**Affiliations:** Department of Periodontology, Semmelweis University, 1088 Budapest, Hungary

**Keywords:** dental implant, peri-implant keratinized mucosa, strip graft, porcine dermal matrix, attached mucosa, peri-implantitis

## Abstract

**Background:** Ensuring a minimum peri-implant keratinized mucosa width (PIKM-W) is critical for maintaining dental implant health, as inadequate PIKM-W is associated with increased risks of plaque accumulation, mucosal inflammation, and peri-implantitis. While epithelialized connective tissue grafts (ECTGs) are considered the gold standard for soft tissue augmentation, they often lead to significant patient morbidity. Xenogeneic dermal matrices (XDMs) offer a less invasive alternative, but are prone to shrinkage, particularly in the mandible. The aim of this study was to evaluate a new surgical method to overcome these limitations with the combination of a narrow band of ECTG (autogenous strip graft, ASG) and an XDM to augment the PIKM-W in the posterior mandible. **Methods:** Twelve patients with a PIKM-W of less than 2 mm in the mandible underwent peri-implant soft tissue augmentation using this combined approach. Changes in the PIKM-W were measured preoperatively; immediately postoperatively; and at 1, 3, 6, 9, and 12 months. Graft remodeling (shrinkage or contraction) and PIKM thickness (PIKM-T) were also evaluated over time. **Results:** Preoperatively, the mean PIKM-W was 0.39 ± 0.40 mm and the PIKM-T was 1.36 ± 0.43 mm. At 6 months, the mean PIKM-W was 4.93 ± 0.98 mm and the PIKM-T was 2.88 ± 0.80 mm, with shrinkage of 39.2 ± 14.1%. By 12 months, the mean PIKM-W stabilized at 4.58 ± 1.28 mm and the PIKM-T stabilized at 2.83 ± 0.65 mm, with shrinkage of 42.2% ± 16.8%. **Conclusions:** There were statistically significant differences in clinical parameters between the baseline and 6 and 12 months (*p* < 0.05). This technique demonstrated the potential for stable augmentation of PIKM-W and PIKM-T over time, with manageable shrinkage. However, further studies with larger sample sizes are needed to confirm its clinical efficacy as an alternative for mandibular keratinized mucosa augmentation around implants.

## 1. Introduction

There has been an ongoing debate in the literature regarding the necessity of a minimum peri-implant keratinized mucosa width (PIKM-W) to maintain peri-implant health [1,2]. Studies have suggested that an inadequate PIKM-W may lead to poor plaque control, mucosal inflammation, and peri-implant tissue recession, all of which may contribute to long-term attachment loss and peri-implantitis [3,4,5,6,7,8]. In the literature, there seems to be a consensus that a 2 mm wide band of keratinized mucosa is clearly beneficial to maintaining adequate peri-implant health [8,9].

Various surgical methods have been introduced to enhance peri-implant keratinized tissues when PIKM-W is insufficient. Based on systematic reviews, the combination of apically positioned partial thickness flaps (APPTFs) and epithelialized connective tissue grafts (ECTGs) can be considered the gold standard technique for achieving an increase in the PIKM-W [10,11,12]. Among the various graft materials, ECTG is preferred due to its stable long-term results and minimal shrinkage [13,14,15]. However, the use of an ECTG is associated with significant patient morbidity and esthetic challenges, particularly color mismatch between grafted tissues and surrounding areas [16,17].

In recent years, clinicians have explored alternative materials, such as xenogeneic collagen matrices (XCMs) and xenogeneic dermal matrices (XDMs), to reduce patient morbidity while maintaining acceptable clinical outcomes [18,19,20,21]. A porcine-derived XCM previously showed promising results in increasing the PIKM-W; however, its high rate of postoperative shrinkage limits its long-term effectiveness [19,20]. In contrast, a porcine-derived XDM demonstrated a greater resistance to shrinkage than the XCM, which may make it a more reliable option for restoring the PIKM-W, even in difficult areas [22,23,24,25].

Despite its advantages, the use of an XDM alone has been associated with successful outcomes in the maxilla, but has presented less predictability in the mandible. A recent prospective case series published by our group found that applying an XDM in the maxilla effectively restored the PIKM-W, achieving a mean PIKM-W of 2.36 mm ± 1.34 mm at the 12-month follow-up. In contrast, applying an XDM in the mandible did not predictably result in a sufficient PIKM-W at the 12-month follow-up, averaging at 1.08 mm ± 1.07 mm [24]. This difference might be due to a shallower vestibule presented at the mandible and a more coronal adhesion of buccal muscles, leading to increased contraction and compromised graft stability.

Recognizing these challenges, the combination of autogenous grafts with xenogeneic materials was suggested to overcome the disadvantages of each approach when used independently. Han et al. introduced the concept of using autogenous strip graft (ASG) as a narrow band of ECTG to augment insufficient PIKM-W by minimizing donor site morbidity and promoting rapid healing [26]. Urban et al. later refined this approach by using a gingival ASG in combination with an XCM to increase the PIKM-W in the anterior maxilla, demonstrating significant improvements in both tissue stability and esthetic outcomes [27,28,29]. This combination leverages the complementary benefits of both materials: an apically placed ASG acts as a mechanical barrier, and it promotes cellular migration and differentiation, while an XCM reduces patient morbidity and improves the esthetic results of the procedure [29].

However, the posterior mandible presents unique anatomical challenges, such as a shallower vestibule and coronal adhesion of buccal muscles, which contribute to higher rates of graft contraction and less predictable outcomes. These challenges complicate grafting procedures and result in poorer clinical outcomes compared to the maxilla. While the combination of ASG and XCM has shown promising outcomes in the anterior maxilla, its effectiveness in the posterior mandible has not been thoroughly evaluated. This represents a significant gap in the literature, as no studies to date have specifically assessed the combined use of ASG and xenogeneic dermal matrices (XDMs) in this challenging anatomical region.

Therefore, the primary aim of this study was to evaluate the clinical outcomes achieved with the combined application of an ASG and an XDM for the reconstruction of the PIKM-W around dental implants in the posterior mandible.

## 2. Materials and Methods

### 2.1. Study Design

This case series aimed to investigate the clinical outcomes of twelve patients with an inadequate PIKM-W (<2 mm) in the mandible. All the participants were treated with an APPTF and the combination of ASG + XDM, and they were followed up for a period of 12 months. The patients were treated at the Department of Periodontology, Semmelweis University, Budapest, Hungary. Prior to surgery, all the participants provided written informed consent. The study was approved by the Semmelweis University Regional and Institutional Committee of Science and Research Ethics (approval number: SE RKEB 223/2017). The research was conducted with full adherence to the principles outlined in the Declaration of Helsinki, as revised in 2013 [30].

### 2.2. Eligibility Criteria

Inclusion Criteria:

- Age ≥ 18 years;
- Patients with at least one dental implant in the posterior mandible that had been in function for more than three months;
- PIKM-W of less than 2 mm, measured using a UNC-15 periodontal probe;
- Good compliance with follow-up protocols and willingness to participate in long-term maintenance programs;
- Good oral hygiene, with a full-mouth plaque score (FMPS) of less than 20%;
- The absence of uncontrolled or untreated periodontal disease, with a full-mouth bleeding score (FMBS) greater than 20%;

Exclusion Criteria:

- Active infectious diseases (HBV, HCV, HIV, TB, SARS-CoV-2, etc.);
- Ongoing chemotherapy or radiation therapy;
- Radiation therapy involving the cranial region within the past 2 years;
- Uncontrolled diabetes;
- Clinically significant osteoporosis or other systemic disease involving bone metabolism;
- Clinically significant circulatory disorders including decompensated cardiac failure;
- Haemodynamically significant valvular heart failure or myocardial infarction within the last 3 months;
- Clinically significant coagulopathy;
- Ongoing or previous systemic corticosteroid therapy within the past 2 months;
- Ongoing or previous systemic bisphosphonate therapy;
- Alcohol or drug abuse;
- Smoking;
- Pregnant or lactating women.

### 2.3. Presurgical Treatment

All patients received professional oral hygiene instructions and full-mouth supragingival scaling prior to surgery. In cases where signs of peri-implant inflammation were present, non-surgical treatment was performed at least six weeks prior to the surgery. Additionally, 400 mg of ibuprofen was administered before the procedure.

### 2.4. Surgical Treatment

**Figure 1:**
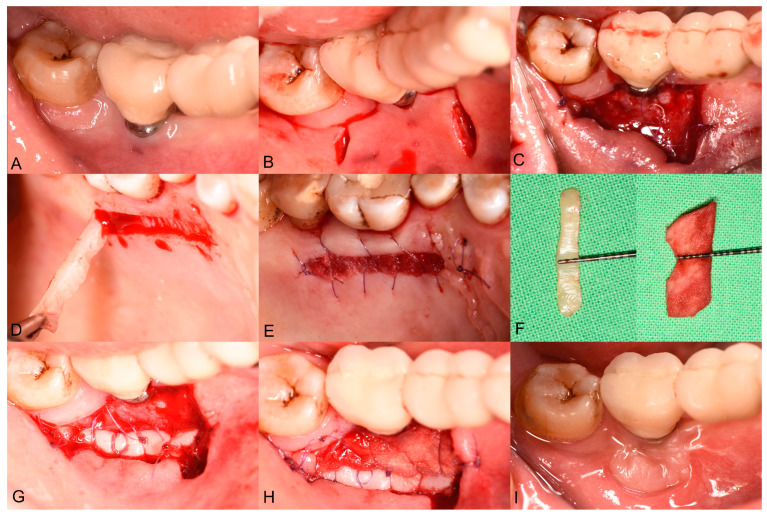
Surgical steps of palatal strip graft (ASG) combined with XDM. (**A**) Buccal view of the insufficient PIKM-W after implant placement. (**B**) A horizontal incision was performed along the mucogingival junction (MGJ), followed by two vertical supraperiosteal incisions at the mesial and distal aspects. (**C**) The coronal flange of the buccal mucosal flap was immobilized to its apical portion using a continuous suturing technique. (**D**) The ASG was harvested from the hard palate. (**E**) A xenogenic collagen matrix strip was sutured over the donor site. (**F**) XDM was trimmed and rehydrated with sterile saline and ASG was harvested. (**G**) The ASG was secured apically using single interrupted sutures. (**H**) Both XDM and ASG were secured with deep periosteal internal horizontal mattress sutures. (**I**) The 12-month follow-up.

Surgeries were carried out by an experienced periodontal specialist (AH). Following local anesthesia at the recipient site, a horizontal incision was made along the mucogingival junction (MGJ) using a 15C blade. Two vertical supraperiosteal incisions were then created at the mesial and distal aspects of the edentulous area (Figure 1B). A split-thickness flap was prepared in an apical direction from the MGJ using either a blade or a periosteal elevator, creating a recipient periosteum bed to accommodate the graft material. The split-thickness mucosal flap was then positioned and secured apically onto the underlying periosteum (Figure 1C).

An autogenous strip graft (ASG) was harvested from the palate under local anesthesia (Figure 1D). An approximately 2–3 mm wide ASG was harvested (Figure 1), with its length adjusted to match the mesiodistal extension of the recipient bed. After applying pressure to the donor site using sterile gauze, a bovine-derived absorbable collagen fleece (Lyostypt, B. Braun, Rubí, Spain) was placed and secured with 6-0 non-resorbable sling sutures (Chiraflon, Chirmax, Prague, Czech Republic) (Figure 1E). The ASG was then positioned on the apical aspect of the recipient bed (Figure 1G) and secured using single interrupted sutures with a 6-0 resorbable monofilament suturing material (Monolac, Chirmax, Prague, Czech Republic). Thereafter, an XDM (mucoderm^®^, Botiss, Zossen, Germany) was sized (Figure 1F) to fit the remaining periosteal bed between the apically placed ASG. The XDM was fixed with single interrupted sutures using a 6-0 resorbable monofilament suturing material. Lastly, deep periosteal internal horizontal mattress sutures were placed to compress both the ASG and the XDM on the periosteum bed, using 5-0 monofilament non-resorbable sutures (Figure 1H). The sutures were removed 14 days postoperatively.

### 2.5. Postsurgical Instructions and Infection Control

After the surgical procedures, the patients were instructed to avoid mechanical cleaning at the surgical sites for two weeks. Instead, chemical plaque control was recommended, using an oral antiseptic containing 0.12% chlorhexidine and 0.05% cetylpyridinium chloride (Paroex, GUM Sunstar, Etoy, Switzerland) twice daily. The patients were prescribed 250 mg amoxicillin combined with 125 mg clavulanic acid (Augmentin 375, GlaxoSmithKline, Brentford, UK), which were to be taken three times daily in the first postoperative week. Pain management was tailored to individual needs, with diclofenac 50 mg (Cataflam 50, Novartis, Basel, Switzerland) prescribed as needed. Follow-up visits were scheduled at 1–2 weeks and at 6–12 months post surgery to monitor plaque control and to assess the healing process.

### 2.6. Outcomes

The primary outcome of this study was the overall change in buccal PIKM-W. The PIKM-W was assessed at baseline (preoperatively); directly after surgery; and at 1-, 3-, 6-, 9-, 12-month follow-ups. The PIKM-W was measured in millimeters using a UNC-15 periodontal probe, assessing the distance from the mid-buccal margin of the implant to the MGJ.

The secondary outcomes were changes in the peri-implant keratinized tissue thickness (PIKM-T) and dimensional changes (shrinkage or contraction) in the grafts. The PIKM-T was measured 2 mm apically from the marginal mucosa using a needle equipped with a rubber stop. Both measurements were rounded to the nearest millimeter. The PIKM-T was evaluated at baseline (preoperatively) and at 1-, 3-, 6-, 9-, 12-month follow-ups. The shrinkage of the grafts is expressed as a percentage between the direct postoperative measurements and the corresponding follow-up timepoint.

### 2.7. Statistical Analysis

The mean, standard deviation, minimum, maximum, and median were calculated for each study variable. All variables are presented as mean ± standard deviation. Statistical significance was tested using inferential statistics, with a significance level of *p* = 0.05. The normality of the examined variables was assessed using the Shapiro–Wilk test. As all variables met normality assumptions, parametric statistical tests were performed for evaluation. Differences in PIKM-W and PIKM-T between the various time points were assessed using repeated-measures ANOVA. Additionally, effect sizes (partial η^2^) and 95% confidence intervals (CIs) were calculated to provide a more comprehensive evaluation of the magnitude and clinical relevance of observed differences. For PIKM-W, the repeated-measures ANOVA resulted in a partial η^2^ = 0.90 (95% CI: [0.76, 0.94]), indicating an extremely large effect. For PIKM-T, the effect size was partial η^2^ = 0.44 (95% CI: [0.34, 0.54]), representing a strong effect. Differences between the 6- and 12-month follow-up data were also analyzed separately using Student’s paired sample *t*-test, with effect sizes and confidence intervals reported to better assess clinical significance. All statistical calculations were performed using the STATA software package (release 18; StataCorp LLC, College Station, TX, USA).

## 3. Results

### 3.1. Patient Demographics

A total of twelve patients were included in this case series, with a mean age of 59.4 years (range: 51–70 years). The cohort consisted of 11 female patients and one male patient. The included 12 patients presented 12 bone level dental implants, seven of which were located on the left side and five on the right side. All patients underwent an APPTF + ASG + XDM intervention for peri-implant keratinized mucosa reconstruction. No complications or adverse events were reported during the 12-month follow-up period by any of the patients.

### 3.2. Primary Outcome: Changes in PIKM-W

**Figure 2:**
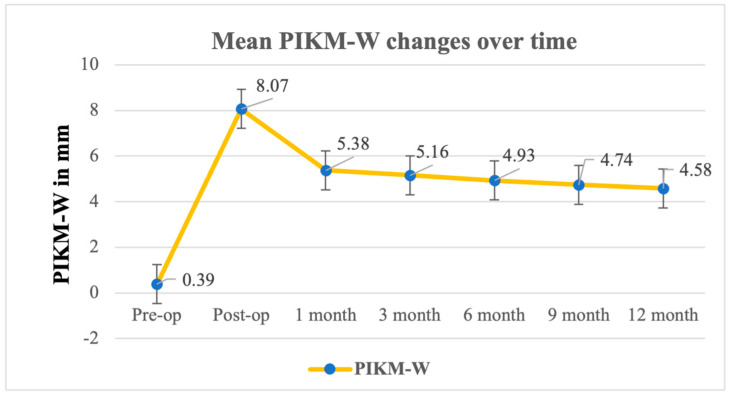
Twelve-month follow-up on PIKM-W alterations after augmentation.

The primary outcome was the change in the PIKM-W between several key timepoints: baseline; immediately after surgery (postoperatively); and at the 1- 3-, 6-, 9-, and 12-month follow-ups (Figure 2). The mean PIKM-W changed from 0.39 mm ± 0.40 mm at baseline to 4.93 mm ± 0.98 mm at the 6-month follow-up and to 4.58 mm ± 1.28 mm at the 12-month follow-up (Table 1). The repeated-measures ANOVA results showed that the differences in the PIKM-W measured were statistically significant (F (6, 66) = 96.86, *p* < 0.0001). The repeated-measures ANOVA indicated a significant change in PIKM-W over time (F (6, 66) = 96.86, *p* < 0.0001), with a very large effect size (η^2^_p_ = 0.90, 95% CI: [0.76, 0.94]). The difference between the 6- and 12-month follow-ups was evaluated separately, and no statistically significant difference could be observed between the two mean values (Table 2) (*p* = 0.0644).

### 3.3. Secondary Outcome: PIKM-T, Graft Remodeling

**Figure 3:**
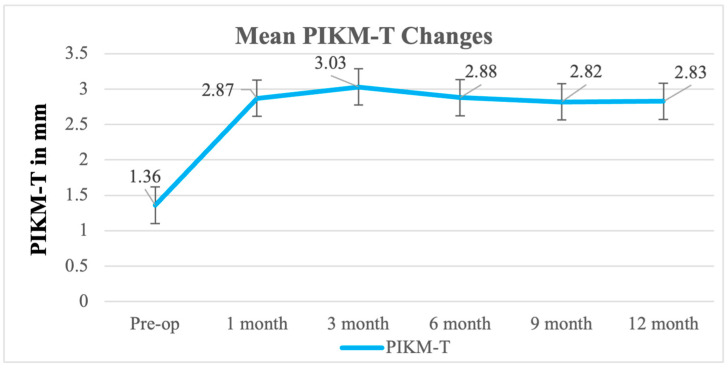
Changes over time in the thickness of keratinized tissue.

The mean PIKM-T values were 1.36 mm ± 0.43 mm, 2.88 mm ± 0.80 mm, and 2.93 mm ± 0.65 mm at baseline, 6-month follow-up, and 12-month follow-up, respectively (Figure 3). Similarly to the PIKM-W values, the PIKM-T also showed a statistically significant difference between the timepoints (F (5, 54) = 12.83, *p* < 0.0001). Similarly, PIKM-T showed a significant change over time (F (5, 54) = 12.83, *p* < 0.0001), corresponding to a large effect size (η^2^_p_ = 0.44, 95% CI: [0.34, 0.54]). However, the 6- and 12-month follow-up PIKM-T values were found to be statistically non-significant (*p* = 0.8439) (Table 3).

**Figure 4:**
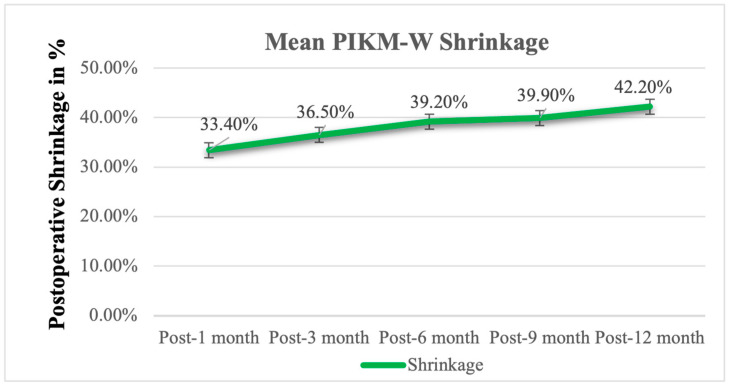
Postoperative evolution of PIKM-W shrinkage.

The postoperative shrinkage of the PIKM-W of the graft was evaluated and is shown in Figure 4. At the 3-month follow-up, the graft decreased in width by 36.5% ± 14.3%, and by the 6-month follow-up, shrinkage increased to 39.2% ± 14.1%, which continued to increase to 42.2% ± 16.8% by the 12-month follow-up (Table 4).

## 4. Discussion

The present study aimed to evaluate the effectiveness of an APPTF combined with the simultaneous use of an ASG and an XDM in increasing the PIKM-W around dental implants in the posterior mandible. The participants were followed over the course of a 12-month period to assess graft remodeling. The results show significant improvements in the PIKM-W at the 12-month follow-up, with the mean PIKM-W being 4.58 mm ± 1.28 mm, which, based on the literature data, is sufficient to ensure proper peri-implant tissue health [31,32,33].

Accounting for contraction, it was necessary to oversize the XDM compared with the desired final outcome. The initial mean postoperative PIKM-W was 8.07 mm ± 1.43 mm, which showed a gradual dimensional contraction of about 36,5% within the first three postoperative months (resulting in a 5.16 ± 0.95 mm PIKM-W at the 3-month follow-up). The rate of dimensional shrinkage gradually reduced over time, and, between the 3- and 6-month follow-ups, it was only about 3%, which was maintained for the remaining follow-up period. By the 12-month follow-up, the PIKM-W stabilized at 4.74 ± 1.11 mm, reflecting a total shrinkage of 42.2% compared with the immediate postoperative measurements.

While this contraction may seem substantial, it is consistent with previous studies on soft tissue augmentation. Urban et al. and Tavelli et al. reported comparable or greater shrinkage rates in xenogeneic and autogenous graft-based augmentation procedures [15,28]. Importantly, despite this remodeling, the final keratinized mucosa width remained above the clinically critical threshold of 2 mm, which has been established as essential for maintaining peri-implant tissue health [9]. Additionally, our previous study using XDM alone demonstrated an 87.4% shrinkage rate, further supporting that the combination of a strip graft with XDM significantly improves graft retention and reduces contraction [24]. Furthermore, the observed stabilization of PIKM-W between 6 and 12 months (*p* = 0.0644) suggests that further shrinkage beyond this timeframe is unlikely. These findings align with the existing literature, confirming the predictability and long-term stability of the strip graft + XDM approach for peri-implant soft tissue augmentation [27,28,29].

These findings suggest that the majority of graft remodeling occurs within the first three postoperative months, after which graft dimensions stabilize. This gradual reduction over time is typical of graft remodeling, where tissue contracts as part of the wound-healing process but stabilizes after the initial healing phase. These results are consistent with the findings of Urban et al., who observed significant graft shrinkage in the early months following surgery, with sufficient keratinized mucosa maintained for long-term peri-implant stability. In the aforementioned study, the overall shrinkage was about 43%, with the PIKM-W reducing from 11.07 mm ± 3.10 mm post surgery to 6.33 mm ± 2.16 mm by the 12-month follow-up [28,29]. However, the current results were found to be more desirable compared to our previous study, where the overall shrinkage was 87.4% at 12 months when using an XDM alone [24]. The results of the current and previous studies suggest that the combination of an ASG and an XDM may offer more resistance to contraction than an XDM alone [28,29]. The degree of graft shrinkage observed in this study did not compromise the final clinical outcomes.

When comparing the results of this study to previous investigations, it is important to highlight that not only the material but also the surgical technique could play a crucial role in clinical success, particularly in the challenging posterior mandible. Previous studies have demonstrated the challenges of graft contraction and unpredictable outcomes when using XDM alone in the mandible [24]. According to the authors’ knowledge, this is the first prospective case series to evaluate the combined use of an ASG and an XDM for peri-implant soft tissue augmentation in the mandible, with a follow-up period of 12 months. This novel approach addresses the anatomical and functional challenges unique to the mandible, such as higher rates of graft contraction and unpredictable outcomes [28,29]. These findings emphasize the need for further research to evaluate how this combined technique can be optimized to achieve even more stable outcomes over time.

The combined use of an ASG and an XDM could provide several advantages, since the use of the gold standard ECTG alone is often associated with higher patient morbidity, increased discomfort at the donor site, and potential for poor esthetic outcomes due to color mismatch [20]. By utilizing a combined approach, our study aimed to minimized patient morbidity and discomfort and to achieve favorable esthetic results. Patient-centered outcomes were not investigated in detail due to the small sample size.

The superior clinical outcomes observed with the combined ASG + XDM technique, compared to XDM alone, can be attributed to several factors. The autogenous strip graft (ASG) may serve as a biomechanical barrier that stabilizes the apical region of the recipient bed, preventing the rebound of alveolar mucosa. This stabilization mechanism has been previously demonstrated in studies utilizing xenogeneic matrices for soft tissue augmentation. Additionally, the ASG provides a robust scaffold for cellular migration and differentiation, enhancing tissue integration and keratinized mucosa formation, as highlighted by Urban et al. [27,28,29].

Furthermore, the XDM contributes to wound stability by reducing shrinkage and providing a biocompatible, porous structure that facilitates the infiltration of fibroblasts and vascular components [22,23,24]. This synergy between ASG and XDM likely explains the enhanced performance of the combined approach in achieving wider and thicker keratinized mucosa.

While this study provides valuable insights into the effectiveness of ASG + XDM, some limitations must be addressed. One of the main limitations is the relatively short follow-up period (12 months) and the small sample size (*n* = 12), which restricts the generalizability of our findings. However, as a prospective case series, this study was designed to provide preliminary evidence regarding the feasibility of the strip graft + XDM technique. Although longer-term follow-up is necessary to confirm the durability of the outcomes, our data indicate that graft contraction stabilizes after 6 months (*p* = 0.0644 between 6 and 12 months), suggesting that further shrinkage beyond this timeframe is unlikely.

Additionally, case series inherently lack the statistical power required to establish causality or make definitive comparisons with alternative techniques. While larger-scale comparative studies would offer a more robust assessment, the novelty of this technique necessitated an initial exploratory study to justify further investigations. Future research should focus on conducting randomized controlled trials (RCTs) with direct comparisons between Strip + XDM and conventional techniques, such as epithelialized connective tissue grafts (ECTGs), to further validate our findings.

Another key limitation is the absence of a control group. While a direct comparison with an established technique (e.g., ECTGs) would provide a clearer evaluation of efficacy, this study was designed as an exploratory investigation to assess the feasibility and clinical performance of Strip + XDM. Given the promising results observed, future studies should incorporate a randomized controlled design to compare Strip + XDM with the gold standard ECTG and assess its long-term efficacy.

## 5. Conclusions

Within the limitations of this study, the application of the ASG + XDM technique showed promise in increasing the PIKM-W in the mandible. At the 12-month follow-up, the PIKM-W remained above the clinically desired 2 mm. The findings align with the existing literature, suggesting that the most pronounced graft shrinkage occurs within the first 3 months of healing, followed by a gradual stabilization. While these results indicate potential benefits of ASG + XDM, further large-scale, randomized clinical trials are required to validate its efficacy and long-term outcomes.

## Figures and Tables

**Table 1:** Peri-implant keratinized mucosa width (PIKM-W) changes over time (mm).

	Baseline	Postop	1 Month	3 Months	6 Months	9 Months	12 Months
Mean	0.39	8.07	5.38	5.16	4.93	4.74	4.58
St. Dev. ^1^	0.40	1.43	0.93	0.95	0.98	1.11	1.28
Min.	0.00	5.00	4.00	3.70	3.50	3.00	6.70
Max.	1.00	10.00	7.00	6.70	6.70	3.00	7.00
Median	0.40	8.15	5.00	5.00	4.85	5.00	4.50
*p*-value ^2^	F (6, 66) = 96.86; *p* < 0.0001; partial η^2^ = 0.90 (95% CI: [0.76, 0.94]						

^1^ Standard deviation. ^2^ Repeated-measures ANOVA.

**Table 2:** Differences in peri-implant keratinized mucosa width (PIKM-W) during follow-ups.

Primary Outcome: Peri-Implant Keratinized Mucosa Width (PIKM-W)			
Difference (Δ)	Patients (*n* = 12)		
	Δ	SD	*p*-Value
Preop-1m	4.98	1.05	<0.0001
Preop-3m	4.77	1.03	<0.0001
Preop-6m	4.53	1.11	<0.0001
Preop-9m	4.35	1.33	<0.0001
Preop-12m	4.19	1.45	<0.0001

**Table 3:** Peri-implant keratinized mucosa thickness (PIKM-T) changes over time.

	Baseline	1 Month	3 Months	6 Months	9 Months	12 Months
Mean	1.36	2.87	3.03	2.88	2.82	2.83
St. Dev. ^1^	0.43	0.82	0.74	0.80	0.59	0.65
Min.	1.00	2.00	2.00	1.50	2.00	2.00
Max.	2.00	5.00	4.00	4.00	4.00	4.00
Median	1.15	2.85	3.00	3.00	3.00	2.75
*p*-value ^2^	F (5, 54) = 12.83, *p* < 0.0001; partial η^2^ = 0.44 (95% CI: [0.34, 0.54]					

^1^ Standard deviation. ^2^ Repeated-measures ANOVA.

**Table 4:** Graft shrinkage over time following surgical intervention (%).

PIKM-W	Postop-1M	Postop-3M	Postop-6M	Postop-9M	Postop-12M
Mean	33.4%	36.5%	39.2%	39.9%	42.2%
SD	13.3%	14.3%	14.1%	16.0%	16.8%
*p*	<0.0001	<0.0001	<0.0001	<0.0001	<0.0001

## Data Availability

Data are contained within the article.
